# Effects of Contrast Media on Renal Function Following Computed Tomography Prior to Transcatheter Aortic Valve Implantation

**DOI:** 10.3390/jcm14248754

**Published:** 2025-12-10

**Authors:** Edward Itelman, Jenan Awesat, Pablo Codner, Yaron Aviv, Tzlil Grinberg, Merry Abitbol, Gideon Shafir, Keren Skalsky, Alon Shechter, Ran Kornowski, Ashraf Hamdan

**Affiliations:** 1 Cardiology Department, Rabin Medical Center, Petah Tikva 4941492, Israel; 2 Grey Faculty of Medical and Health Sciences, Tel Aviv University, Tel Aviv 6997801, Israel; gideons@clalit.org.il; 3 Radiology Department, Rabin Medical Center, Petah Tikva 4941492, Israel

**Keywords:** computed tomography, contrast-induced acute kidney injury, TAVI, chronic kidney disease, renal function, iodinated contrast

## Abstract

**Background:** Preprocedural contrast-enhanced computed tomography (CT) is essential for planning Transcatheter Aortic Valve Implantation (TAVI), but concerns remain regarding contrast-induced (CI) acute kidney injury, especially in patients with chronic kidney disease. This study aimed to evaluate the incidence of CI-acute kidney injury following contrast-enhanced CT performed before TAVI. **Methods:** CI-acute kidney injury was defined according to the Kidney Disease—Improving Global Outcomes (KDIGO) guidelines. Nonionic, low-osmolality iodinated contrast material was used for all CT studies. The primary outcome was the incidence of CI-acute kidney injury post-CT. Secondary outcomes included the need for renal replacement therapy and the 30-day post-CT mortality rate. **Results:** Our study included 359 patients. The median age was 81, and 44% were males. Chronic kidney disease was present in 59.3% of patients, and the overall incidence of CI-acute kidney injury occurred in 24 patients (7%), without a significant difference between patients with and without baseline chronic kidney disease (4.8% vs. 8%, respectively; *p* = 0.331). Three consecutive creatinine tests within a median of 5.8 days showed that acute kidney injury occurred in only six patients (1.7%). No patients required new dialysis initiation within 30 days. Multivariable analysis did not identify baseline chronic kidney disease or IV contrast volume as independent predictors of CI-acute kidney injury. **Conclusions:** CI-acute kidney injury following pre-TAVI CT is low, even among patients with chronic kidney disease. Most cases were transient and did not require dialysis. Routine avoidance of CT due to chronic kidney disease may not be warranted.

## 1. Introduction

Transcatheter Aortic Valve Implantation (TAVI) has become an established treatment for severe aortic stenosis in patients from all risk groups [1,2]. Patients undergoing TAVI are often elderly and commonly have chronic kidney disease, which makes them vulnerable to renal complications [3,4]. Pre-procedural contrast-enhanced computed tomography (CT) angiography is a routine procedure performed for TAVI planning. It is the gold-standard tool for annular sizing, assessing the risk of annular injury and coronary occlusion, determining the optimal coplanar fluoroscopic implantation angle, and evaluating peripheral vascular access. Nonetheless, the intravenous (IV) administration of iodinated contrast in this elderly population has been causally associated with the development of contrast-induced (CI) acute kidney injury, particularly among individuals with preexisting renal dysfunction [5,6,7].

Although some animal studies and clinical reports support the existence of CI-acute kidney injury, the causal relationship between the administration of intravenous iodinated contrast and CI-acute kidney injury remains controversial for several reasons [5,6].

The widely used “multiple-insult animal model” has limited applicability to human renal disease, as it often involves exaggerated nephrotoxic stimuli that do not reflect clinical scenarios [7]. Much of the existing literature on CI-acute kidney injury is derived from invasive angiographic studies involving intra-arterial contrast administration [8].

Few studies have evaluated the risk of CI-acute kidney injury following IV administration of iodinated contrast medium. The reported incidence ranges from 4% to 12% [9,10,11], although some studies have found no significant difference in CI-acute kidney injury rates between patients undergoing CT with or without IV iodinated contrast [12]. Consequently, the relationship between IV contrast administration for noninvasive CT imaging and the true incidence of CI-acute kidney injury remains inadequately characterized, especially among elderly patients with severe symptomatic aortic stenosis in the TAVI era.

Given the conflicting evidence on this topic and the potential risk of withholding crucial pre-procedural imaging, we aimed to evaluate the real-world impact of pre-TAVI CT imaging on renal function and the incidence of CI-acute kidney injury in consecutive patients with severe aortic stenosis.

## 2. Methods

### 2.1. Study Population

**Figure 1:**
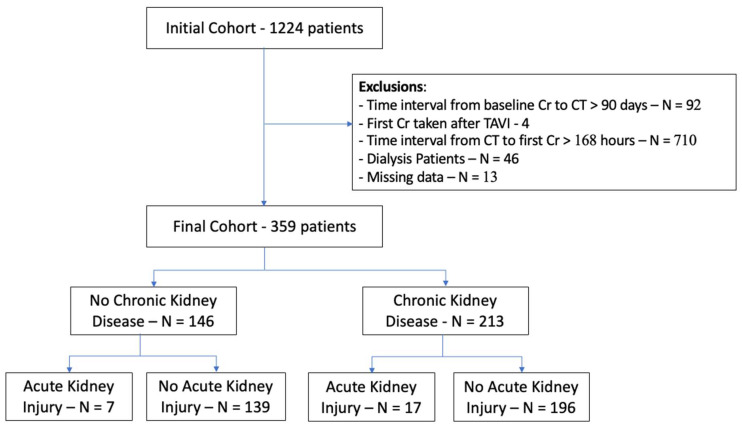
Patient cohort.

This is a retrospective cohort study that included consecutive patients aged 18 years or older who underwent a pre-procedural contrast-enhanced CT before TAVI at our center between 2010 and 2025. The study is based on systematically collected and thoroughly reviewed data from CT reports and electronic medical records of all patients. Patients were excluded if they lacked baseline creatinine measurements within 30 days before the CT scan or follow-up creatinine within 30 days after the CT (Figure 1). Patients who were already undergoing dialysis at the time of the CT scan were excluded; all other patients were included in the study, regardless of their baseline GFR. The Institutional Review Board of our center approved this study, ensuring strict maintenance of participant anonymity through database analyses. No individual consent was required.

### 2.2. Contrast-Enhanced Computed Tomography Pre-TAVI

CT scans were performed using a 256-slice system (Brilliance iCT, Philips Healthcare, Cleveland, OH, USA) or a dual-source CT system (SOMATOM Force, Siemens Healthineers, Forchheim, Germany, with a collimation of 2 × 192 × 0.6 mm and a gantry rotation speed of 250 ms). Tube output was weight-adapted (tube voltage 70–140 kV, tube current range from 450 to 700 mAs). A CT scan was performed using a 256-slice system with a gantry rotation time of 330 ms. Data were collected with a collimation of 96 × 0.625 mm, a tube current of 485 mA at 100 kV, and a pitch value of 0.2. The acquisition was performed during an inspiratory breath hold, with simultaneous electrocardiogram (ECG) recording to enable retrospective data gating.

An intravenous injection of 50 to 70 mL of nonionic low-osmolarity contrast agent (Iopromide 350 mg/mL; Bayer Schering, Berlin, Germany) was administered at a flow rate of 3.5 mL/s, followed by a 40 mL saline chase bolus at the same flow rate. Preprocedural hydration with 500 mL of 0.9% saline was administered to patients with eGFR below 30 mL/min/1.73 m^2^ before undergoing the CT scan. Diuretics were not stopped before the exam for fear of heart failure exacerbation. According to the institutional policy, creatinine measurements were routinely performed in all patients within 3 months before and within 4 weeks after performing the CT scan. In diabetic patients on metformin treatment, the drug was suspended 48 h before the CT examination.

### 2.3. Clinical Data and Study Endpoint

The patient’s baseline demographic and clinical data were carefully retrieved from the computerized medical records. Clinical data were available for the complete cohort. Diagnoses were based on computerized hospitalization records (International Classification of Diseases, Ninth Revision [ICD-10] codes), laboratory tests, and medications. The primary outcome of the current study, Contrast-Induced Acute Kidney Injury (CI-AKI), defined based on the definition for acute kidney injury by the Kidney Disease—Improving Global Outcomes (KDIGO) guidelines. CI-AKI was characterized as either a relative increase in serum creatinine concentration of at least 0.3 mg/dL within 48 h of contrast medium administration compared with baseline, or an increase in serum creatinine to ≥1.5 times the baseline, presumed to have occurred within the prior 7 days [13]. For this purpose, we took three consecutive exams and applied the acute kidney injury criteria to all three; if any one exam met the criteria, the patient was deemed to have suffered acute kidney injury. AKI was considered positive if it met the criteria for any of the three tests in this study. Baseline chronic kidney disease was defined by a prior diagnosis of chronic kidney disease mentioned in the medical record or a baseline eGFR < 60 mL/min/1.73 m^2^. Chronic kidney disease stages were determined based on KDIGO: Stage G1 (GFR ≥ 90), G2 (60–89), G3a (45–59), G3b (30–44), G4 (15–29), and G5 (<15 mL/min/1.73 m^2^) [13]. Secondary outcomes were defined as the initiation of renal replacement therapy within 30 days post-CT and all-cause mortality. Mortality data were available for all subjects from the National Population Register.

### 2.4. Statistical Analysis

Continuous variables were expressed as mean ± standard deviation if normally distributed, or median with interquartile range if skewed. Categorical variables were presented as frequency (%). Continuous data between the study groups (patients with and without chronic kidney disease) were compared with Student’s *t*-test, and categorical data were compared with the chi-square or Fisher’s exact test. Univariate linear regression models were used to determine the unadjusted odds ratio (OR) for the primary and secondary endpoints. Univariate analysis was performed to assess the impact of important clinical factors, including age, sex, prior chronic kidney disease, congestive heart failure, diabetes mellitus, and the amount of IV iodinated contrast medium administered, on the occurrence of CI-acute kidney injury. A multivariate analysis combining all of the above was also performed. Spearman′s Correlation was used to assess the relationship between the volume of contrast medium administered during the CT scan and changes in creatinine values from baseline to subsequent measurements. Sample size calculations were performed to ensure significant power to the study. All statistical analyses were conducted using R (R Foundation for Statistical Computing, Vienna, Austria). An association was considered statistically significant if the two-sided *p* value was less than 0.05.

## 3. Results

### 3.1. Study Population

A total of 359 patients who underwent a pre-TAVI CT were included in this study (Table 1). The median age for the overall cohort was 81 years [IQR 75–85], and 44.3% were male. Baseline chronic kidney disease was common in our cohort, affecting 213 patients (59.3%). This included five patients (1.4%) in stage G5, 30 (8.4%) in stage G4, and 74 (20.6%) in stage G3b. 22 patients (10.3%) had a preexisting diagnosis of CKD with baseline GFR in this sample >60 (Table 1). The median eGFR at baseline was 57 [IQR 41–76] mL/min/1.73 m^2^. The volume of iodinated contrast media injected for each CT scan was 55 ± 10 mL; patients with chronic kidney disease received equivocal volumes of contrast compared to patients without chronic kidney disease (55 ± 10 vs. 54 ± 10 mL, respectively; *p* = 0.255). Baseline creatinine was obtained at a mean of 7 ± 15 days before the CT scan. The first creatinine measurement after the test, at a median of 0.9 (IQR 0.75–2.8) days after the scan, showed no significant change from baseline (*p* = 0.08). The second repeat was measured at a median of 3.7 days (IQR 1.83–9.79) and also revealed no significant difference from the baseline creatinine (*p* = 0.29). Third-repeat creatinine was measured at a median of 5.9 days (IQR 2.85–26) after the CT and also did not differ significantly from baseline creatinine (*p* = 0.13). The most common comorbidities included hypertension (91%) and dyslipidemia (93%), which were more prevalent among patients with baseline chronic kidney disease (84% vs. 95%, *p* = 0.001) and 88% vs. 97% *p* = 0.002). Prevalence of atrial fibrillation −45% in the general cohort also differed significantly among groups (36% vs. 51%, *p* = 0.01). Diabetes mellitus (60%), COPD (23%), and peripheral vascular disease (26%) did not differ significantly amongst groups (*p* = 0.90, *p* = 0.93, and *p* = 0.24, respectively). Patients with chronic kidney disease were older than patients without chronic kidney disease (median age 82 vs. 78 years, respectively, *p* < 0.001). However, there was no significant difference in sex distribution between the groups (44% males vs. 45%, respectively, *p* = 0.972) (Table 1). Table 2 shows a stratification of the study population based on the primary study endpoint—any acute kidney injury.

### 3.2. Study Endpoints

**Figure 2:**
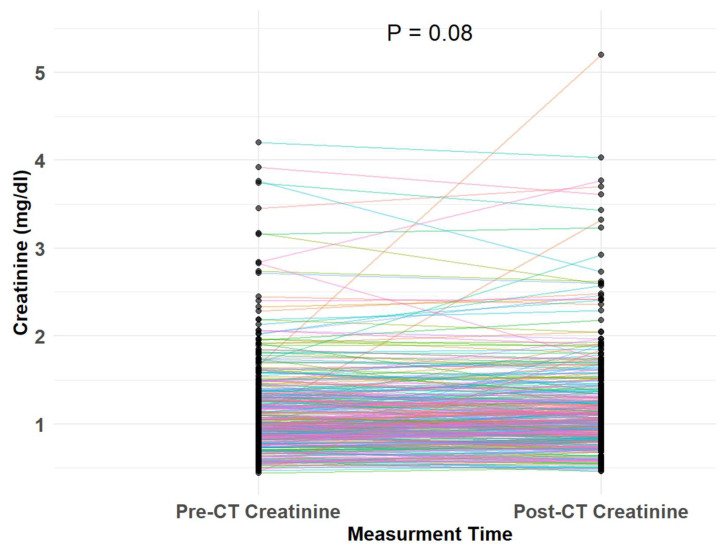
Comparison of baseline and after CT createnine measurement.

**Figure 3:**
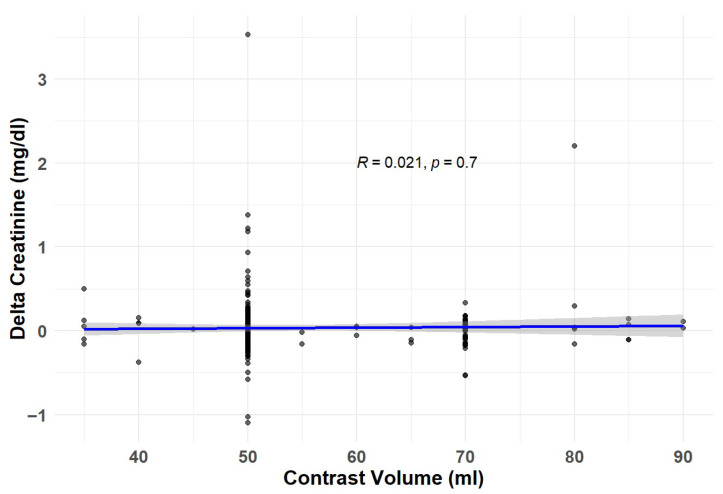
Relationship between Contrast Amount and Change in Creatinine.

**Figure 4:**
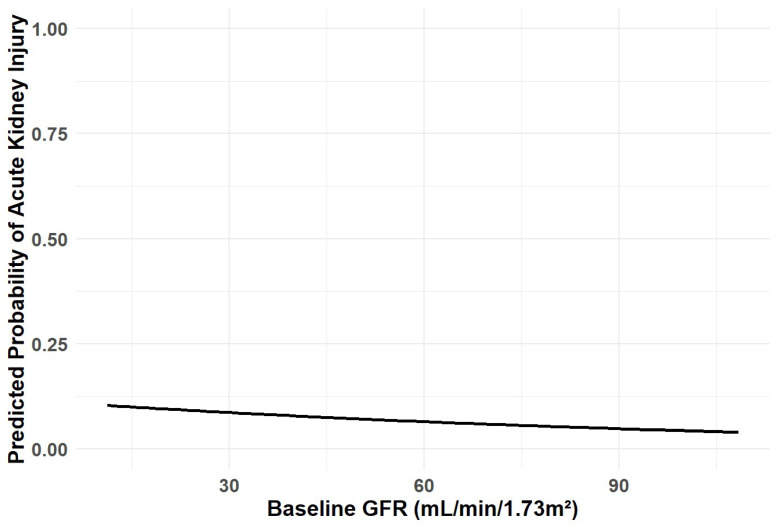
Predicted Probability of Acute Kidney Injury by Baseline GFR.

Of the 359 included patients, 24 (6.7%) developed CI-acute injury following the CT scan during the entire follow-up period. There was no significant difference in creatinine measurements pre- and post-CT (Figure 2). There was no statistically significant difference in the incidence of CI-acute kidney injury between patients with and without chronic kidney disease [17/213 (8%) vs. 7/146 (5%), respectively, *p* = 0.331] (Table 3). Notably, Spearman’s rank correlation analysis revealed no significant association between the volume of iodinated contrast media administered and the change in serum creatinine level (rho = 0.021, *p* = 0.7), Figure 3. Figure 4 presents a predictive model illustrating the probability of CI-acute kidney injury as a function of baseline eGFR. No significant association was observed between eGFR and the likelihood of CI-acute kidney injury (*p* = 0.288). In univariate analysis, baseline chronic kidney disease status, age, sex, diabetes mellitus, and amount of contrast media were not associated with CI-acute kidney injury (Table 4). In a multivariate analysis adjusted for baseline chronic kidney disease status, age, sex, diabetes, and the amount of contrast media, none of these variables were associated with CI-acute injury (Table 4).

During the 30-day follow-up, no patients required new dialysis therapy. Three deaths (0.8%) occurred within one week of the CT scan, all in patients with symptomatic critical aortic stenosis. One death occurred in a non-chronic kidney disease patient, and two deaths occurred in patients with baseline chronic kidney disease; the difference was not statistically significant (*p* = 0.1).

## 4. Discussion

ECG-triggered CT plays an important role in the workup of patients referred for TAVI and is therefore a routine preprocedural study. However, the high prevalence of chronic kidney disease among TAVI candidates has raised concerns about CI-acute kidney injury. Our study evaluated the real-world impact of preprocedural contrast-enhanced CT on renal function in patients undergoing CT before TAVI. Overall renal function remained largely stable after the CT scan, with no statistically significant change in serum creatinine between pre-CT and post-CT measurements. As defined by KDIGO criteria, CI-acute kidney injury occurred in only a small subset of patients (6.7%), with no significant difference between patients with and without baseline chronic kidney disease. No significant difference in creatinine from baseline was observed across three consecutive tests, and no patient required new dialysis within 30 days. These findings are consistent with several recent studies in other clinical settings that have found no major deterioration in renal function attributable solely to IV iodinated contrast media administration [6,7].

In line with our study, a systematic review and meta-analysis by McDonald et al. of controlled studies comparing patients exposed to IV iodinated contrast medium with patients who underwent an imaging examination without contrast medium [14] revealed no difference in the incidence of CI-acute kidney injury, dialysis, and death in patients who receive IV contrast medium and those who do not, including subgroups of patients with renal insufficiency. These results suggest that there may be no increased risk of acute kidney injury associated with IV contrast medium administration. Acute kidney injury that occurred after a CT scan may instead be attributed to contrast medium-independent factors [11].

Studies conducted across various CT settings provide conflicting evidence on the role of iodinated contrast injection in the development of acute kidney injury. One study conducted in patients undergoing CT to diagnose pulmonary embolism used propensity-matching to control for important confounders and found that IV contrast material administration was not associated with an increased risk of acute kidney injury in patients with suspected pulmonary embolism [15]. Another study performed on patients suffering from sepsis performed on 4171 patients found that the incidence of acute kidney injury was 7.2% in those who underwent contrast-enhanced CT, 9.4% in those who underwent CT with no contrast enhancement, and 9.7% in those who underwent no CT. In this study, contrast administration was not associated with an increased incidence of acute kidney injury. Finally, A large meta-analysis found that contrast-enhanced CT was not significantly associated with either acute kidney injury (odds ratio (OR) 0.94; 95% confidence interval (CI) 0.83–1.07), need for renal replacement therapy (OR 0.83; 95% CI 0.59–1.16), or all-cause mortality (OR 1.0; 95% CI 0.73–1.36) [16].

A previous study of TAVI patients reported higher rates of post-CT CI-acute kidney injury than those observed in our study. Jochheim et al. [17] reported CI-acute kidney injury rates of up to 10.5% in a cohort of 361 patients. Their study found that individuals with chronic kidney disease (eGFR < 60 mL/min/1.73 m^2^) were more prone to CI-acute kidney injury (81.6% vs. 64.4%, *p* = 0.045), potentially due to the administration of higher volumes of iodinated contrast media in these patients—a trend noted in their analysis. Consistent with our findings, most patients recovered renal function after the CT scan. However, the applicability of Jochheim et al.′s results to current patient populations may be limited. The study, published in 2014, was conducted during a period when TAVI was primarily reserved for high-surgical-risk patients [15]; accordingly, those who developed CI-acute kidney injury had higher EuroSCORE II values than those who did not. Furthermore, the use of relatively large contrast volumes (91 ± 29 mL) likely influenced their outcomes. Notably, contrast volumes >90 mL were associated with a significantly increased risk of CI-acute kidney injury. With recent advances in imaging protocols and technology, lower contrast volumes are now feasible. Our study utilizes an average of 55 ± 10 mL.

Our findings apply only to IV administration of iodinated contrast media. Intra-arterial administration of contrast media for angiographic procedures has been reported to be associated with a higher incidence of CI-acute kidney injury, dialysis, and mortality, as compared with IV contrast administration [18,19]. It is uncertain whether these higher incidences are attributable to the route of administration, differences in patient susceptibility among those undergoing invasive procedures, or to iatrogenic causes arising from the procedures themselves, such as hemodynamic changes during the procedures or atherosclerotic embolization [20].

Our study also aimed to evaluate the risk of CI-acute kidney injury in relation to baseline eGFR. A predictive model was constructed to plot the probability of CI-acute kidney injury across the spectrum of baseline eGFR values (Figure 3). While the curve suggested a trend toward slightly higher CI-acute kidney injury rates with decreasing eGFR, this association did not reach statistical significance (*p* = 0.288). Numerically, the predicted risk of CI-acute kidney injury declined from approximately 10% at eGFR levels below 30 mL/min/1.73 m^2^ to about 5% at values above 90 mL/min/1.73 m^2^. Despite a qualitatively higher predicted risk among those with lower eGFR, the effect size was modest, and the confidence intervals overlapped substantially across eGFR strata, indicating no significant association between baseline renal function and CI-acute kidney injury risk.

Several factors may explain our cohort’s relative preservation of kidney function. First, contemporary iodinated contrast agents are associated with reduced nephrotoxicity compared to earlier high-osmolar formulations. Second, individuals with preexisting chronic kidney disease often received intensified care, including prophylactic IV fluids (particularly in those with eGFR <30 mL/min/1.73 m^2^) and close laboratory monitoring during follow-up, further minimizing the likelihood of renal injury.

## 5. Limitations

Due to the study’s single-center, retrospective design, our findings may reflect local clinical features and may limit generalizability. In addition, for this specific patient collective (median age 82 years), no robust data or guidelines recommend a unique hydration protocol before contrast media injection. Thus, the length and amount of IV hydration used before the CT scan in the present patients reflect local management. It is also important to note that although creatinine tests were performed consecutively, the IQR of the third test varied from 2.8 to 26 days. Furthermore, a large proportion of patients in the initial cohort screened for this trial did not have serum creatinine measured within the first week after the CT exam, reflecting local clinical practice. While more frequent daily testing might have identified a higher incidence of early contrast-associated AKI, it likely would not have altered the recovery rates observed in our study.

## 6. Conclusions

In this retrospective study of TAVI candidates undergoing preprocedural contrast-enhanced CT, intravenous administration of iodinated contrast medium did not significantly impair renal function in this subset of patients, including those with preexisting chronic kidney disease. While a small subset developed CI-acute kidney injury, the overall incidence was low and temporary, and no patients required dialysis. These findings suggest that concerns about CI-acute kidney injury may be less pronounced than previously thought.

## Figures and Tables

**Table 1:** Demographic and Clinical Features—stratified by Chronic Kidney Disease.

Variable	Overall	NO-CKD	CKD	*p*
Number of patients	359	146	213	
Age, years (median [IQR])	81 (75–85)	78 (73–84)	82 (77–86)	<0.001
Male sex, n (%)	159 (44.3)	64 (43.8)	95 (44.6)	0.972
Baseline eGFR (median [IQR])	57 (41–78)	78 (72–87)	45 (36–54)	<0.001
Chronic kidney disease present, n (%)	213 (59.3)	0 (0.0)	213 (100.0)	<0.001
CKD stage distribution, n (%)				<0.001
Stage G5	5 (1.4)	0 (0.0)	5 (2.3)	
Stage G4	30 (8.4)	0 (0.0)	30 (14.1)	
Stage G3b	74 (20.6)	0 (0.0)	74 (34.7)	
Stage G3a	82 (22.8)	0 (0.0)	82 (38.5)	
Stage G2	143 (39.8)	123 (84.2)	20 (9.4)	
Stage G1	25 (7.0)	23 (15.8)	2 (0.9)	
Hypertension, n (%)	325 (90.5)	123 (84.2)	202 (94.8)	0.001
Congestive heart failure, n (%)	215 (59.9)	77 (52.7)	138 (64.8)	0.029
Chronic obstructive pulmonary disease, n (%)	84 (23.4)	35 (24.0)	49 (23.0)	0.932
Peripheral vascular disease, n (%)	94 (26.2)	33 (22.6)	61 (28.6)	0.248
Stroke/TIA, n (%)	183 (51.0)	79 (54.1)	104 (48.8)	0.381
Dyslipidemia, n (%)	334 (93.0)	128 (87.7)	206 (96.7)	0.002
Diabetes mellitus, n (%)	214 (59.6)	86 (58.9)	128 (60.1)	0.907
Atrial fibrillation, n (%)	161 (44.8)	53 (36.3)	108 (50.7)	0.01
ASA treatment, n (%)	186 (51.8)	77 (52.7)	109 (51.2)	0.854
P2Y12 treatment, n (%)	57 (15.9)	16 (11)	41 (19.2)	0.05
OAC treatment, n (%)	207 (57.7)	70 (47.9)	137 (64.3)	0.003
RAASi treatment, n (%)	138 (38.4)	42 (28.8)	96 (45.1)	0.003
Beta Blockers treatment, n (%)	260 (72.4)	93 (63.7)	167 (78.4)	0.003
Statin treatment, n (%)	281 (78.3)	99 (67.8)	182 (85.4)	<0.001

Continuous data are shown as mean ± SD (if normally distributed) or median [IQR]; categorical data are shown as n (%).

**Table 2:** Demographic and Clinical Features—stratified by Acute Kidney Injury.

Variable	Overall	NO-AKI	AKI	*p*
Number of patients	359	335	24	
Age, years (median [IQR])	80.73 [74.90, 85.28]	80.68 [74.79, 84.98]	81.51 [77.93, 87.09]	0.275
Male sex, n (%)	159 (44.3)	147 (43.9)	12 (50.0)	0.711
Baseline eGFR, (median [IQR])	56.74 [41.37, 77.50]	57.50 [41.87, 77.93]	48.65 [40.20, 68.50]	0.25
Chronic kidney disease present, n (%)	213 (59.3)	196 (58.5)	17 (70.8)	0.331
CKD stage distribution, n (%)				0.612
Stage G5	5 (1.4)	5 (1.5)	0 (0.0)	
Stage G4	30 (8.4)	26 (7.8)	4 (16.7)	
Stage G3b	74 (20.6)	68 (20.3)	6 (25.0)	
Stage G3a	82 (22.8)	77 (23.0)	5 (20.8)	
Stage G2	143 (39.8)	136 (40.6)	7 (29.2)	
Stage G1	25 (7.0)	23 (6.9)	2 (8.3)	
Hypertension, n (%)	325 (90.5)	303 (90.4)	22 (91.7)	1
Congestive heart failure, n (%)	215 (59.9)	197 (58.8)	18 (75.0)	0.178
Chronic obstructive pulmonary disease, n (%)	84 (23.4)	77 (23.0)	7 (29.2)	0.659
Peripheral vascular disease, n (%)	94 (26.2)	85 (25.4)	9 (37.5)	0.287
Stroke/TIA, n (%)	183 (51.0)	173 (51.6)	10 (41.7)	0.464
Dyslipidemia, n (%)	334 (93.0)	310 (92.5)	24 (100.0)	0.331
Diabetes mellitus, n (%)	214 (59.6)	196 (58.5)	18 (75.0)	0.169
Atrial fibrillation, n (%)	161 (44.8)	149 (44.5)	12 (50.0)	0.754
ASA treatment, n (%)	186 (51.8)	169 (50.4)	17 (70.8)	0.086
P2Y12 treatment, n (%)	57 (15.9)	54 (16.1)	3 (12.5)	0.857
OAC treatment, n (%)	207 (57.7)	190 (56.7)	17 (70.8)	0.255
RAASi treatment, n (%)	138 (38.4)	132 (39.4)	6 (25)	0.236
Beta Blockers treatment, n (%)	260 (72.4)	240 (71.6)	20 (83.3)	0.317
Statin treatment, n (%)	281 (78.3)	261 (77.9)	20 (83.3)	0.714

Continuous data are shown as mean ± SD (if normally distributed) or median [IQR]; categorical data are shown as n (%).

**Table 3:** Study Endpoints.

Variable	Overall	NO-CKD	CKD	*p*
Number of patients	359	146	213	
Any acute kidney injury, n (%)	24 (6.7)	7 (4.8)	17 (8.0)	0.331
Contrast volume injected, mL (mean ± SD)	54 ± 10	54 ± 10	54 ± 10	0.255
New dialysis within 30 days, n (%)	0 (0)	0 (0)	0 (0)	–
Mortality within one week of CT, n (%)	3 (0.8)	1 (0.6)	2 (0.9)	1

Continuous data are shown as median [IQR]; categorical data are shown as n (%).

**Table 4:** Predictors of Acute Kidney Injury.

		Univariate Analysis			Multivariate Analysis	
	OR	95% CI	*p*-Value	OR	95% CI	*p*-Value
Chronic kidney disease	1.72	0.72–4.56	0.240	1.46	0.59–3.97	0.430
Age (per year)	1.04	0.98–1.10	0.223	1.02	0.97–1.09	0.432
Male	1.28	0.55–2.96	0.561	1.14	0.48–2.70	0.764
Diabetes Mellitus	2.13	0.87–5.99	0.119	2.00	0.80–5.72	0.159
Contrast injected	0.97	0.91–1.02	0.254	0.97	0.91–1.02	0.318

## Data Availability

The original contributions presented in this study are included in the article. Further inquiries can be directed to the corresponding author.
